# Supplementary material for: Role of Climate and Edaphic Factors on the Community Composition of Biocrusts Along an Elevation Gradient in the High Arctic
Source: Microorganisms. 2024 Dec 17;12(12):2606. doi: 10.3390/microorganisms12122606 (PMC11676250; doi:10.3390/microorganisms12122606)
Supplement: Supplementary file 1 [file microorganisms-12-02606-s001.zip › Supplementary_Figures_Ossian.pdf]

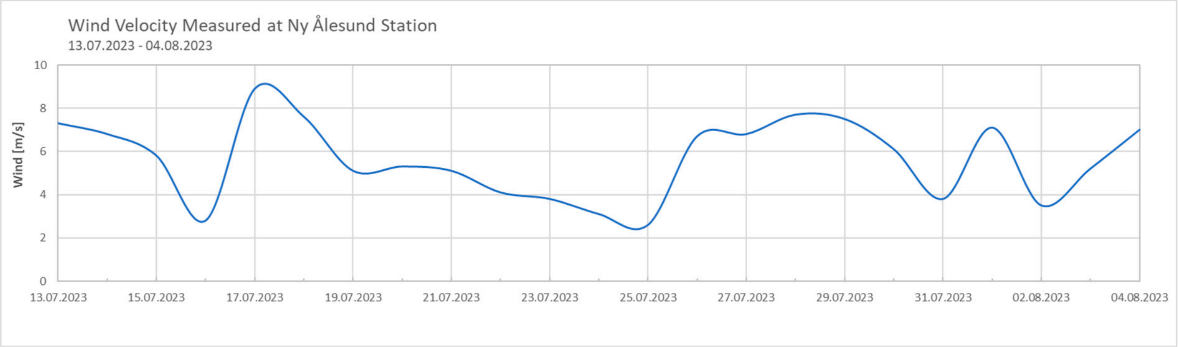

Supplementary Figure S1: Wind velocity in the time period from 13.07.2023 until 04.08.2023 measured at Ny-Ålesund station. Ny-Ålesund station is located around 12 km west from Ossian Sarsfjellet.

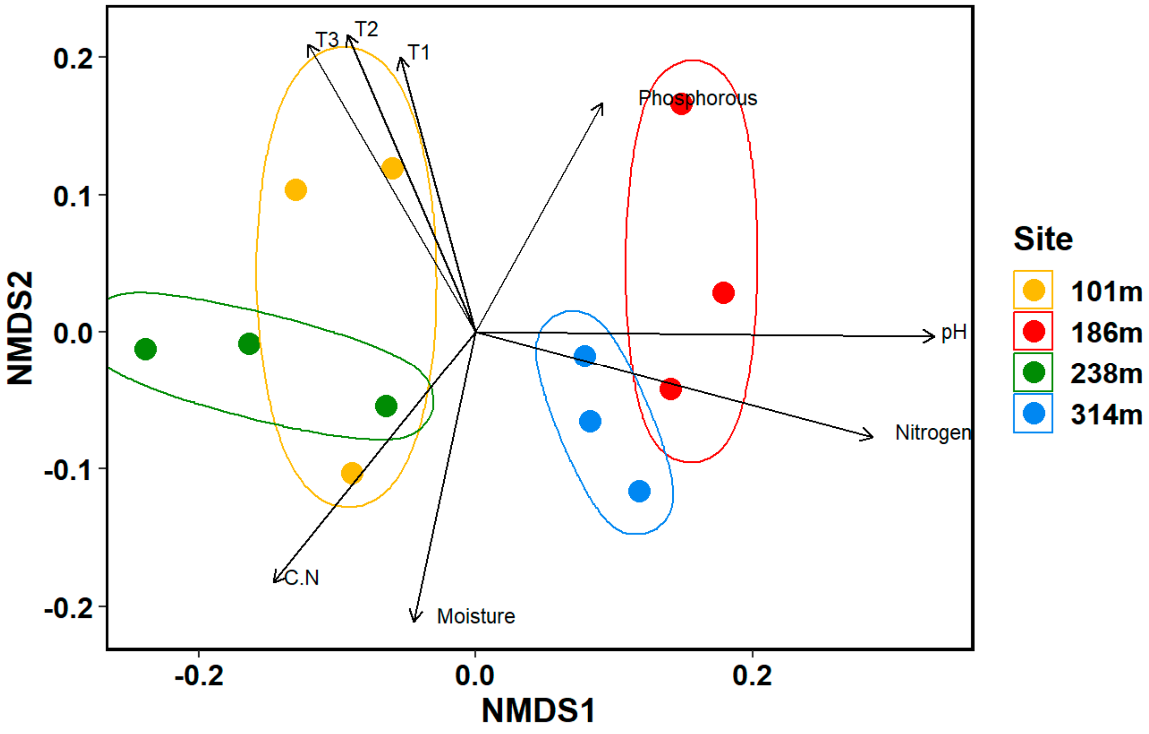

Supplementary Figure S2: Non-Metric Multidimensional Scaling (NMDS) plot based on relative abundances of DNA sequences. T1 = Temperature measured 6 cm below the soil surface; T2 = Temperature measured at the soil surface; T3 = Temperature measured 15 cm above the soil surface; C.N = Carbon to nitrogen ratio; Parameters which did not show significant differences between the sites are not included, p-value < 0.05.
